# Genetic landscape and immune mechanism of monocytes associated with the progression of acute-on-chronic liver failure

**DOI:** 10.1007/s12072-022-10472-y

**Published:** 2023-01-10

**Authors:** Jia Yao, Tian Liu, Qiang Zhao, Yaqiu Ji, Jinjia Bai, Han Wang, Ruoyu Yao, Xiaoshuang Zhou, Yu Chen, Jun Xu

**Affiliations:** 1 Department of Gastroenterology, Shanxi Bethune Hospital, Shanxi Academy of Medical Sciences, Tongji Shanxi Hospital, Third Hospital of Shanxi Medical University, Taiyuan, 030032 China; 2 Department of Biochemistry and Molecular Biology, School of Basic Medicine, Shanxi Medical University, Taiyuan, 030001 China; 3 Department of Nephrology, The Affiliated People’s Hospital of Shanxi Medical University, Taiyuan, 030032 China; 4 Fourth Department of Liver Disease (Difficult and Complicated Liver Diseases and Artificial Liver Center), Beijing You’an Hospital Affiliated to Capital Medical University, Beijing, 100069 China; 5 The First Hospital of Shanxi Medical University, No. 85 Jiefang South Road, Yingze District, Taiyuan, 030032 Shanxi China

**Keywords:** Single-cell RNA sequencing, Acute-on-chronic liver failure, Monocytes, Systemic inflammation, Immune mechanism

## Abstract

**Objective:**

Acute-on-chronic liver failure (ACLF) has a high prevalence and short-term mortality. Monocytes play an important role in the development of ACLF. However, the monocyte subpopulations with unique features and functions in ACLF and associated with disease progression remain poorly understood. We investigated the specific monocyte subpopulations associated with ACLF progression and their roles in inflammatory responses using the single-cell RNA sequencing (scRNA-seq).

**Methods:**

We performed scRNA-seq on 17,310 circulating monocytes from healthy controls and ACLF patients and genetically defined their subpopulations to characterize specific monocyte subpopulations associated with ACLF progression.

**Results:**

Five monocyte subpopulations were obtained, including pro-inflammatory monocytes, CD16 monocytes, HLA monocytes, megakaryocyte-like monocytes, and NK-like monocytes. Comparisons of the monocytes between ACLF patients and healthy controls showed that the pro-inflammatory monocytes had the most significant gene changes, among which the expressions of genes related to inflammatory responses and cell metabolism were significantly increased while the genes related to cell cycle progression were significantly decreased. Furthermore, compared with the ACLF survival group, the ACLF death group had significantly higher expressions of pro-inflammatory cytokines (e.g., IL-6) and their receptors, chemokines (e.g., CCL4 and CCL5), and inflammation-inducing factors (e.g., HES4). Additionally, validation using scRNA-seq and flow cytometry revealed the presence of a cell type-specific transcriptional signature of pro-inflammatory monocytes THBS1, whose production might reflect the disease progression and poor prognosis.

**Conclusions:**

We present the accurate classification, molecular markers, and signaling pathways of monocytes associated with ACLF progression. Therapies targeting pro-inflammatory monocytes may be a promising approach for blocking ACLF progression.

**Supplementary Information:**

The online version contains supplementary material available at 10.1007/s12072-022-10472-y.

## Introduction

Although the definition of acute-on-chronic liver failure (ACLF) differs between the East and the West, the prevalence and short-term mortality of this condition are high in different geographical areas, making it a global burden of disease [1, 2]. It has been recognized that persistent inflammation and immune dysregulation with initial wide-spread immune activation play a central role in the progression of ACLF [3, 4]. However, its specific mechanism is still unclear.

The inflammatory response of monocytes is an important part of the immune system's response to pathogenic invasion. During the acute inflammation of ACLF, the inflammatory response induced by monocytes and their subpopulations may play a pivotal role in ACLF, including the production of inflammatory cytokines, phagocytosis, and production of repair molecules. Thus, monocytes are considered the fuel for inflammation [5–7]. ACLF patients are highly susceptible to infections, and monocytes are the first line of defense against pathogens entering the circulation [8]. Circulating monocytes and tissue-resident macrophages (Kupffer cells) are the main antigen-presenting cells. They play key roles in antigen presentation, pathogen recognition, and phagocytosis and are associated with the development of various inflammatory diseases and the angiogenesis [9]. Monocytes can sense changes in the inflammatory environment, spread inflammation at the systemic level, and trigger abnormal production of immune factors. In addition, under the action of pro-inflammatory and vaso-permeating mediators, monocytes accumulate in the liver, leading to the damage of liver tissue. The damaged liver further activates immune cells and promotes the development of systemic inflammatory responses, thereby leading to disease progression [10]. Dysregulation of this immune response can lead to metabolic disturbances and acute inflammation in ACLF patients, ultimately leading to multiple organ failure and even death. Although monocytes play critical roles in ACLF progression, little is known about the functional heterogeneity of specific monocyte subpopulations in ACLF, and their exact functions, gene expression profiles, and activation status remain unclear. Therefore, we attempted to characterize monocyte subpopulations and their specific gene expression signatures in ACLF in more detail, thereby identifying the causative genes and biomarkers that contribute to ACLF progression.

This study aimed to elucidate the genetic landscape and associated inflammatory response of monocytes during ACLF progression. Single-cell RNA sequencing (scRNA-seq) technology enables the rapid and precise determination of gene expression patterns in thousands of individual cells in an unbiased manner. It clusters subpopulations of cells with different functions, thereby defining accurate taxonomies, identifying subpopulations, and revealing the cellular composition and characteristics of monocytes in ACLF to generate detailed genetic maps. Using this technique, we further explored the heterogeneity among ACLF monocyte subpopulations and the difference in functional phenotypes and elucidated the relationships of monocytes with inflammatory responses and immune activation during ACLF progression, in an attempt to identify new therapeutic targets for the treatment and prognosis of ACLF.

## Patients and methods

### Patients

Six patients diagnosed with HBV-ACLF at the Department of Gastroenterology of our hospital and three age- and gender-matched healthy controls were enrolled (Table 1). The inclusion criteria were: (1) meeting the diagnostic criteria of COSSH-ACLF ^[^ [11]; 2) all ACLF patients were positive for hepatitis B surface antigen (HBsAg) or HBV-DNA. The exclusion criteria were: (1) with comorbid hepatocellular carcinoma and/or other tumors; (2) with comorbid immune system-related diseases, such as autoimmune hepatitis (AIH) and rheumatism; (3) with comorbid hepatitis caused by alcohol, drug, or other viruses; and (4) taking glucocorticoids or immunosuppressive drugs. The study was approved by the Ethics Committee of Shanxi Bethune Hospital affiliated to Shanxi Medical University.

**Table 1:** Characteristics of the subjects included

	Age (years)	Gender	Final state	Baseline liver disease	HBV-DNA level (IU/mL)	TBIL (μmol/L)	INR	ALT (U/L)	AST (U/L)	AST/ALT	ALB (G/L)	GLB (G/L)	A/G	CREA (μmol/L)	MELD score	PaO_2_/FiO_2_(mmHg)
Case 1	42	Male	Survive	Cirrhosis	3.54 × 10^7^	231.9	1.7	1174	519	0.44	30.7	27.4	1.12	54	17.5	433
Case 2	39	Male	Survive	Cirrhosis	2.43 × 10^6^	251.2	2.2	101	105	1.04	31.8	32.3	0.98	54	20.7	452
Case 3	46	Female	Survive	Cirrhosis	1.65 × 10^5^	211.1	1.6	65	106	1.63	32.5	43.6	0.75	79	20.1	428
Case 4	52	Female	Death	Cirrhosis	2.27 × 10^6^	406.2	3.0	74	130	1.76	29.5	24.6	1.2	55	26.2	463
Case 5	41	Male	Death	Cirrhosis	1.83 × 10^5^	372.8	4.5	26	60	2.37	34.9	21.8	1.6	145	39.7	198
Case 6	40	Male	Death	Cirrhosis	2.18 × 10^5^	255.8	3.3	30	23	0.77	45.5	31.5	1.44	79	29.0	477
Healthy control 1	45	Female	–	–	–	8.0	0.9	37	32	0.86	43	25	1.72	59	–	–
Healthy control 2	47	Male	–	–	–	7.9	0.8	27	25	0.93	52	32	1.63	62	–	–
Healthy control 3	43	Male	–	–	–	9.2	1.0	32	28	0.88	46	34	1.35	64	–	–

*TBIL* total bilirubin (normal range: 5–21 μmol/L), *INR* international normalized ratio (normal range: 0.8–1.2), *CREA* creatinine (normal range: 57–97 μmol/L), *ALT* Alanine aminotransferase (normal range: 9–50 U/L), *AST* Aspartate aminotransferase (normal range: 15–40 U/L), *AST/ALT* DeRitis ratio(normal range: 0.8–1.5), *ALB* Albumin (normal range: 40–55 g/L), *GLB* Globulin (normal range: 20 ~ 40 g/L), *A/G* Albumin globulin ratio (normal range: 1.2–2.4), *MELD* Model for end-stage liver disease, *HBV-DNA* level (normal range: < 100 IU/mL), *PaO*_*2*_*/FiO*_*2*_ a ratio of PaO_2_ of arterial oxygen to FiO_2_ (normal range: 400–500 mmHg)

### Preparation of PBMC samples

Peripheral blood (10 ml) was harvested, and peripheral blood mononuclear cells (PBMCs) were isolated using density gradient centrifugation. Delamination from top to bottom was performed after centrifugation: plasma, PBMCs, lymphocyte separation medium, granulocytes, and erythrocytes. The peripheral blood from PBMCs patients was then collected.

### 10X Genomics single-cell transcriptome sequencing

Cell density and viability were determined for single-cell suspensions, and samples with > 90% cell viability were finally selected. The 10X Genomics library was constructed using a single-cell automated preparation system (ChromiumTM Controller) and a ChromiumTM Single Cell 3ʹ Reagent Kit v.2 (10X Genomics, Pleasanton, CA, USA). Sequencing was performed using the MGISEQ-2000 sequencing platform (BGI, Shenzhen, China).

### Single-cell RNA sequencing data prepossessing, gene expression quantification, and cell type determination

RNA reads were aligned to the reference genome (refdata-gex-GRCh38-2020-A) using the STAR software. The expression of each gene per cell was determined using the Cell Ranger software based on UMI counts. A total of 106,723 cells were captured and 214,627 genes were detected in this study library. Data quality control and filtering were performed using the Seurat software. Cells with a gene number of less than 200 or more than 90% of maximum gene count or those with mitochondrial read percentages greater than 15% were excluded from analysis. A total of 83,577 cells entered the final analysis. Hypervariable features were filtered using the Seurat software, and the top 2000 highly variable genes were selected and used for further analysis.

### Unsupervised clustering and visualization

The gene expression matrix of each cell was normalized using the Seurat R package (version 4.2.0) [12], and the genes with the highest variance in the dataset were selected for analysis. A principal component analysis (PCA) was performed to reduce the dimensionality, and a resolution parameter of 0.5 was chosen for the clustering of all cells. The cells were visualized in two dimensions according to their gene expression profiles using the UMAP.

### Annotating of cell clusters

For each cluster, the marker genes were identified using the FindAllMarkers function in the Seurat package. A marker gene was defined as a gene that is significantly overexpressed (being > 0.25 log-fold higher than the mean expression value in the other sub-clusters), and with a detectable expression in > 25% of all cells from the corresponding sub-cluster [13]. Subsequently, the cell clusters were labeled as known cell types in the Cell Marker database [14].

### Flow cytometry staining and analysis

Single-cell suspensions were treated with a human CD14-positive selection kit following the manufacturer's instructions to obtain high-purity monocytes for flow cytometry. According to the manufacturer's recommendations, staining with APC-H7 mouse anti-human CD14 (BD Biosciences) was first performed; after 30 min incubation, the cells were washed twice with phosphate-buffered saline (PBS), followed by intracellular THBS1 staining with BD THBS1 kit (BD Biosciences). After 30 min incubation, the cells were re-suspended in 300 μL PBS after washing twice with wash buffer. Flow cytometry was performed on a BD Fortessa, and data were analyzed using FlowJo v10.1 software.

### GO and KEGG enrichment analyses

GO biological process (http://geneontology.org/) analysis was performed to explore monocytes in ACLF. Specific pathways were further investigated on the Kyoto Encyclopedia of Genes and Genomes (KEGG) database (https://www.kegg.jp/).

### Statistical analysis

One-way ANOVA and Bonferroni correction were performed on the MFI of THBS1 in ACLF survival group and ACLF death group. Statistical analysis was performed using IBM SPSS Statistics software package (version 25), and histograms were drawn using GraphPad Prism 8.0 (GraphPad Software, Inc., La Jolla, CA).

## Results

### scRNA-seq and major cell typing of PBMCs from healthy controls and ACLF patients

**Fig. 1:**
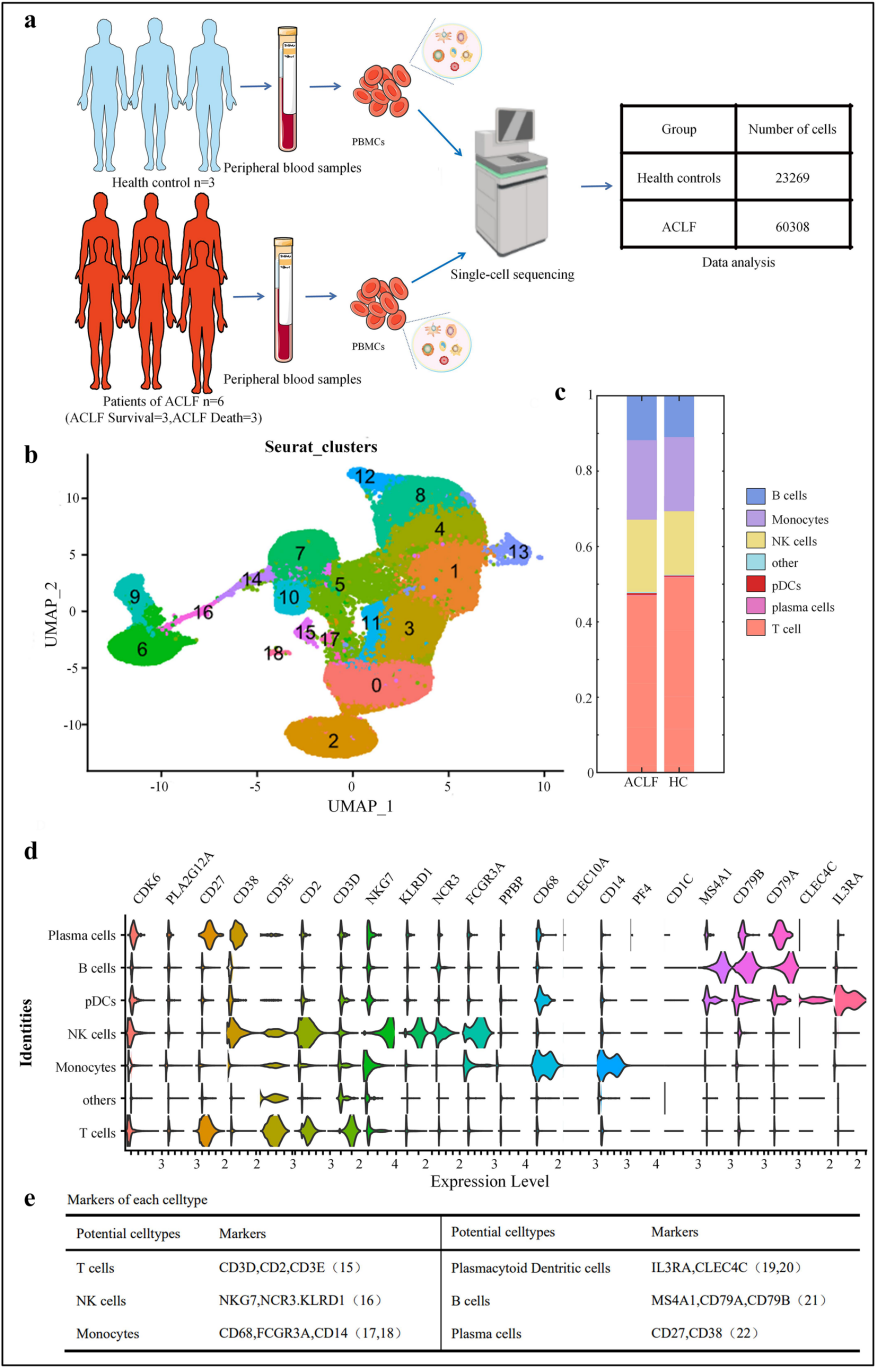
Unsupervised cluster analysis on single-cell RNA-seq data. **A** Work flow of PBMCs isolation and single-cell RNA-Seq. **B** Cell subpopulations detected and their UMAP plots. **C** Proportional bar charts of cell subpopulations in ACLF patients and healthy controls (colors are coded for the cell subpopulations identified in this study). **D** Violin plots showing the expression levels of marker genes for defined cell subpopulations revealed by single-cell sequencing. The distribution of cell subpopulation-specific marker genes across all clusters reflects their cell types. **E** Table shows the marker genes of cell types defined in **D**

We used scRNA-seq to analyze the PBMCs in 3 healthy controls and 6 ACLF patients (including 3 patients in ACLF survival group and 3 in ACLF death group) (Table 1). After strict quality-based screening, 83,577 high-quality sorted cells were obtained, and principal component analysis (PCA) was performed on 2,000 genes with the highest expression variability in these cells (Fig. 1A). Nineteen cell clusters were identified by unsupervised analysis (Fig. 1B). Differential expression analysis was performed among these clusters, and cell subpopulations were defined according to the gene expression patterns of published cell clusters. Consistent with the findings in healthy controls, ACLF patients were also defined as having cell subpopulations corresponding to the six leukocyte types based on differentially expressed genes (DEGs) (Fig. 1C). These cell subpopulations included T cells (clusters 0, 1, 2, 3, and 11) [15], natural killer (NK) cells (clusters 4, 8, 12, and 13) [16], monocytes (clusters 5, 7, 10, 14, and 15) [17, 18], plasmacytoid dendritic cells (cluster 16) [19, 20], B cells (clusters 6 and 9) [21], and plasma cells (cluster 18) [22], and other cellular components (Fig. 1D). To investigate the relationships of monocytes with other clusters, we analyzed pDCs, NK cells, plasma cells, and B cells. In addition, FCGR3A (CD16) was also highly expressed in NK cells. Therefore, combining these marker genes can more accurately identify monocytes. Subsequently, we mainly performed an in-depth analysis of different types of monocytes, with an attempt to explore the differences between ACLF patients and healthy controls in more detail.

Thus, by detecting the gene expression profiles of a large number of single-sorted peripheral blood mononuclear cells (PBMCs), we identified many featured genes of the mononuclear cell population and found that multiple genes were co-expressed in other cellular components of monocytes and other PBMC subsets, suggesting that there may be functional overlap among them.

### Differences in distribution and genotypes among different human monocyte subpopulations

**Fig. 2:**
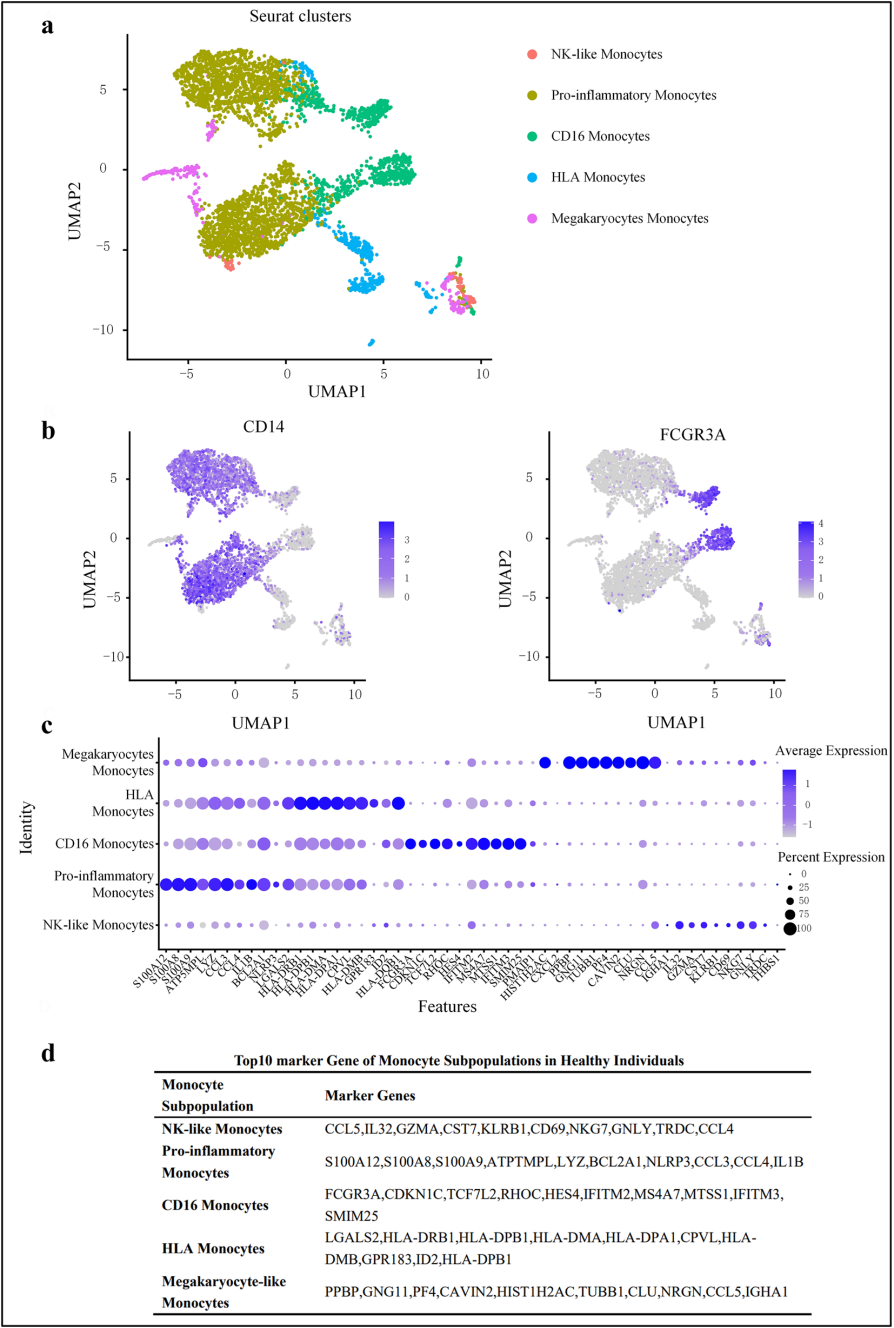
Genotypes and functions of monocyte subpopulations in healthy subjects. **A** UMAP shows transcriptional heterogeneity in circulating monocytes. The 4239 monocytes are further divided into 5 subpopulations, whose names are annotated on the left, and different colors are used to distinguish each cluster. **B** Expression patterns of two marker genes CD14 and FCGR3A (CD16) in human monocytes. Purple represents the expression of these two marker genes and each dot represents an individual cell. **C** Dot plot curve showing the top 10 marker genes revealed by single-cell sequencing of monocyte subpopulations defined in **B**. **D** Table shows the top 10 marker genes of monocyte subpopulations defined in **C**

To further determine the heterogeneity and variation of monocytes at the single-cell level and provide markers for more precise subpopulation classification, we performed scRNA-seq on 4239 circulating monocytes from healthy controls based on the similarities and differences in gene expression profiles. To differentiate the function and activation status of each subpopulation based on differences in gene expression markers, we classified the monocytes into five subpopulations (Fig. 2A). According to the classification method of human monocytes [including classical (CD14^+^CD16^−^), non-classical (CD14^dim^CD16^+^)] [23], we identified classical monocytes with high CD14 expression and non-classical monocytes with high CD16 expression (Fig. 2B). Notably, uniform manifold approximation and projection (UMAP) also showed a monocyte subpopulation expressing both CD14 and CD16 marker genes, which belong to a small subset of CD14 monocytes. Based on the high expression of CD14 and intermediate expression of FCGR3A (CD16), these cells were labeled as intermediate monocytes. Their location on the UMAP may indicate that they are experiencing transition from classical to non-classical monocytes. We further characterized each cluster using the top 10 marker genes (Fig. 2C and D).

The monocytes subpopulations classified in this way exhibited distinct transcriptional and functional characteristics. However, whether all cell subpopulations of these types and other cell subpopulations in the same inflammatory microenvironment have similar functions or whether further sub-classifications exist has not been determined. It was found that pro-inflammatory mediators S100A8 and S100A9 were also highly expressed in a subpopulation with high CD14 expression, so this subpopulation was classified as pro-inflammatory monocytes. In addition, the pro-inflammatory monocytes have unique marker genes including chemokines (CCL3 and CCL4) [9], interleukins (IL-1B), and NLRP3 inflammasome and are closely related with immune regulation, promotion of inflammatory responses, and induction of apoptosis. The non-classical monocytes (CD14^dim^CD16^++^) are a subpopulation with a high expression of FCGR3A (CD16). In addition to the expression of FCGR3A, we also found that the transcription factor TCF7L2, the inflammation-inducible gene HES4, and the interferon-inducible genes IFITM2, IFITM3, MS4A7, MTSS1, and SMIM25 played key roles in the signaling pathways, suggesting that this subpopulation is enriched in genes associated with signal transcription, inflammation induction, and induction of interferon-stimulated antiviral responses. Another subpopulation was HLA monocytes, which expressed the major histocompatibility complex (MHC) class II molecules HLA-DRB1 and HLA-DPB1. While other subpopulations also expressed human leukocyte antigen (HLA)-related genes, the HLA monocytes had the most diverse and highest levels of leukocyte-related gene expressions, including high levels of the unique leukocyte antigens HLA-DMA and HLA-DMB, suggesting that this subpopulation has the strongest antigen processing and presentation capabilities [24]. The gene expression of megakaryocyte-like monocytes was similar to that of megakaryocyte progenitors (including PPBP and PF4), suggesting that megakaryocyte-like monocytes play an important role in activating platelets and mediating chronic inflammation. NK cell-like monocytes expressed genes (e.g., GZMA and GNLY, in addition to KLRB1 and NKG7) similar to those expressed by NK cells, indicating that this subpopulation is involved in cytotoxicity regulation and inflammatory responses and has functional overlap with NK cells.

Thus, through gene expression profiling of PBMCs, we identified five monocyte subpopulations with unique gene expression signatures, which indicated that each monocyte subpopulation has specific functions.

### ACLF monocytes had unique immune characteristics and inflammatory responses

**Fig. 3:**
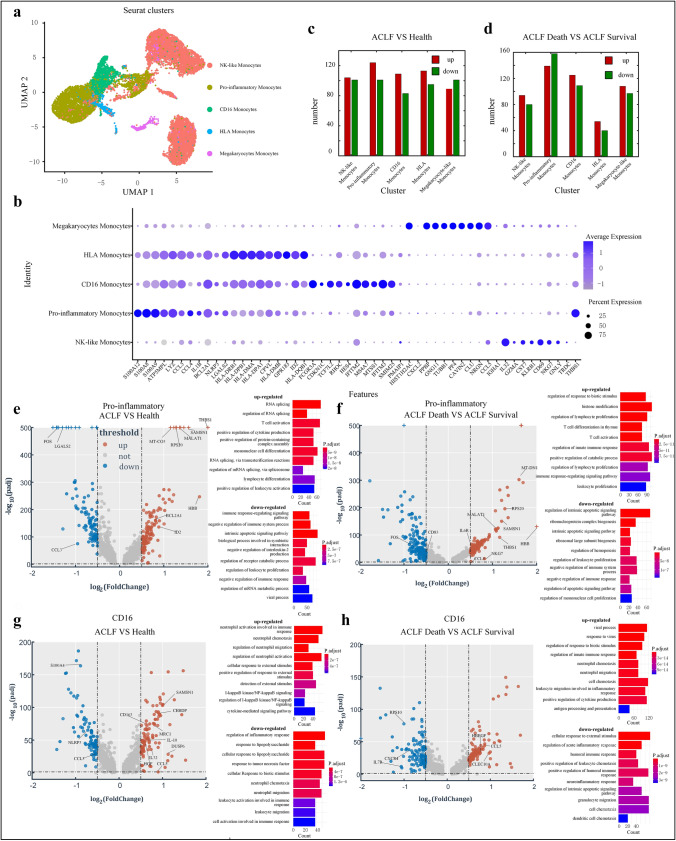
Unique immunological profile of monocytes in ACLF. RNA sequencing was performed on the monocytes of 3 healthy controls and 6 ACLF patients (including 3 patients in the ACLF death group and 3 in the ACLF survival group). **A** The 4239 ACLF monocytes on UMAP are further divided into 5 subpopulations, whose names are annotated on the left, and different colors are used to distinguish each cluster. **B** Dot plot curve showing the top 10 marker genes revealed by single-cell sequencing of ACLF monocyte subpopulations. **C** The number of up- and down-regulated genes per monocyte subpopulation when comparing gene expression changes between ACLF patients (*n* = 6) and healthy controls (*n* = 3). The number of genes with log-fold change (Fc) > 0.5 and adjusted *P* value < 0.05 in each subpopulation were as follows: 104 genes up-regulated and 101 down-regulated in NK-like Monocytes subpopulation; 124 genes up-regulated and 101 down-regulated in pro-like monocytes subpopulation; 109 genes up-regulated and 83 down-regulated in CD16 monocytes subpopulation; 113 genes up-regulated and 95 down-regulated in HLA monocytes subpopulation; 89 genes up-regulated and 101 down-regulated in megakaryocyte-like monocytes subpopulation. **D** The number of up- and down-regulated genes per monocyte subpopulation when comparing gene expression changes between ACLF death group (*n* = 3) and ACLF survival group (*n* = 3). The number of genes with log-fold change (Fc) > 0.5 and adjusted *P* value < 0.05 in each subpopulation were as follows: 94 genes up-regulated and 80 down-regulated in NK-like Monocytes subpopulation; 139 genes up-regulated and 158 down-regulated in Pro-like Monocytes subpopulation; 125 genes up-regulated and 109 down-regulated in CD16 Monocytes subpopulation; 54 genes up-regulated and 40 down-regulated in HLA Monocytes subpopulation; and 108 genes up-regulated and 97 down-regulated in megakaryocyte-like monocytes subpopulation. The volcano plots show all the up-regulated (red) and down-regulated (green) genes in monocytes when: **E.** comparing pro-inflammatory monocytes between healthy controls and ACLF patients; **F** comparing CD16 monocytes between healthy controls and ACLF patients; **G** comparing Pro-inflammatory Monocytes between ACLF death group and ACLF survival group; **H** comparing monocytes between ACLF survival group and ACLF death group. The top 10 biological processes of the differentially expressed genes were identified

Monocytes are associated with ACLF progression, but their exact functions, subpopulations, cellular and molecular characteristics, and activation status in ACLF remain unclear. To identify the heterogeneity and variations in cellular components, we performed scRNA-seq on 13,071 high-quality circulating monocytes from ACLF patients and compared their gene expression patterns with monocytes from healthy controls. In accord with findings in the healthy population, the monocytes from ACLF patients were still clustered into five subpopulations based on the expressions of DEGs (Fig. 3A and B). Abundant evidence suggests that monocytes play an important role in the early stages of disease by mediating both pro- and anti-inflammatory responses. In fact, monocytes are well known for their dual roles in coordinating inflammatory responses and regulating tissue repair [25, 26]. In the present study, Based on the differences in gene expressions among monocyte subpopulations between the healthy controls and the ACLF patients, the most significant difference between up-regulated and down-regulated genes among all monocyte subpopulations was found in pro-inflammatory monocytes, followed by CD16 monocytes (non-classical monocytes) (Fig. 3C). Subsequently, we further subdivided the ACLF group into the ACLF survival group (*n* = 3) and ACLF death group (*n* = 3). The most significant change in gene expression among all monocyte subpopulations was also observed in the pro-inflammatory monocytes (Fig. 3D). Therefore, our analysis focused on pro-inflammatory monocytes and CD16 (FCGR3A) monocytes.

Pro-inflammatory monocytes presented with most significant changes in gene expressions, including up- and down-regulated genes, suggesting that pro-inflammatory monocytes may be actively involved in the development and progression of ACLF. Compared to healthy controls, genes that were more than twice as highly expressed in the ACLF patients included HBB gene, a member of the hemoglobin family, and thrombospondin 1 (THBS-1), a member of the thrombospondin family (THBS). Other up-regulated genes included *SAMSN1*, *MALAT1*, *PRS20*, and *MT-CO3*, which are associated with activation of immune cells and promotion of inflammatory responses [27–29]. Genes down-regulated in the pro-inflammatory monocytes of ACLF patients included FOS, CCL3 and LGALS2, which are mainly associated with resistance to viral infection, promotion of apoptosis, and immunosuppression (Fig. 3E). Compared with the ACLF survival group, the ACLF death group had higher levels of pro-inflammatory cytokines and their receptors (e.g., IL-6R), and cytotoxic factors (e.g., NKG7) (Fig. 3F), further suggesting that increased inflammation may be an influencing factor for ACLF progression and poor prognosis.

The subpopulation with the second largest number of altered genes was CD16 monocytes (non-classical monocytes). Compared with those in the healthy controls, genes up-regulated in the ACLF group included scavenger receptors (CD163 and MRC1), growth factors (e.g., HGF), and inhibitory cytokines (e.g., IL10), suggesting their involvement in anti-inflammatory responses. The down-regulated genes included *NLRP3*, *CCL3*, and S100A4, showing the inflammatory effects of CD16 monocytes (Fig. 3G). In addition, the CD16 monocytes in the ACLF death group had similar biological functions to those in the ACLF survival group (Fig. 3H). Therefore, rather than simply promoting or inhibiting inflammation, CD16 monocytes have dual regulatory effects in apoptotic signaling pathways, inflammatory responses, and cytokine chemotaxis and migration in ACLF.

Therefore, pro-inflammatory monocytes promote inflammatory and immune responses, while CD16 monocytes may play a dual role of promoting and inhibiting inflammation in the occurrence and development of ACLF.

### ACLF progression-related biomarker THBS1 and its pathways

**Fig. 4:**
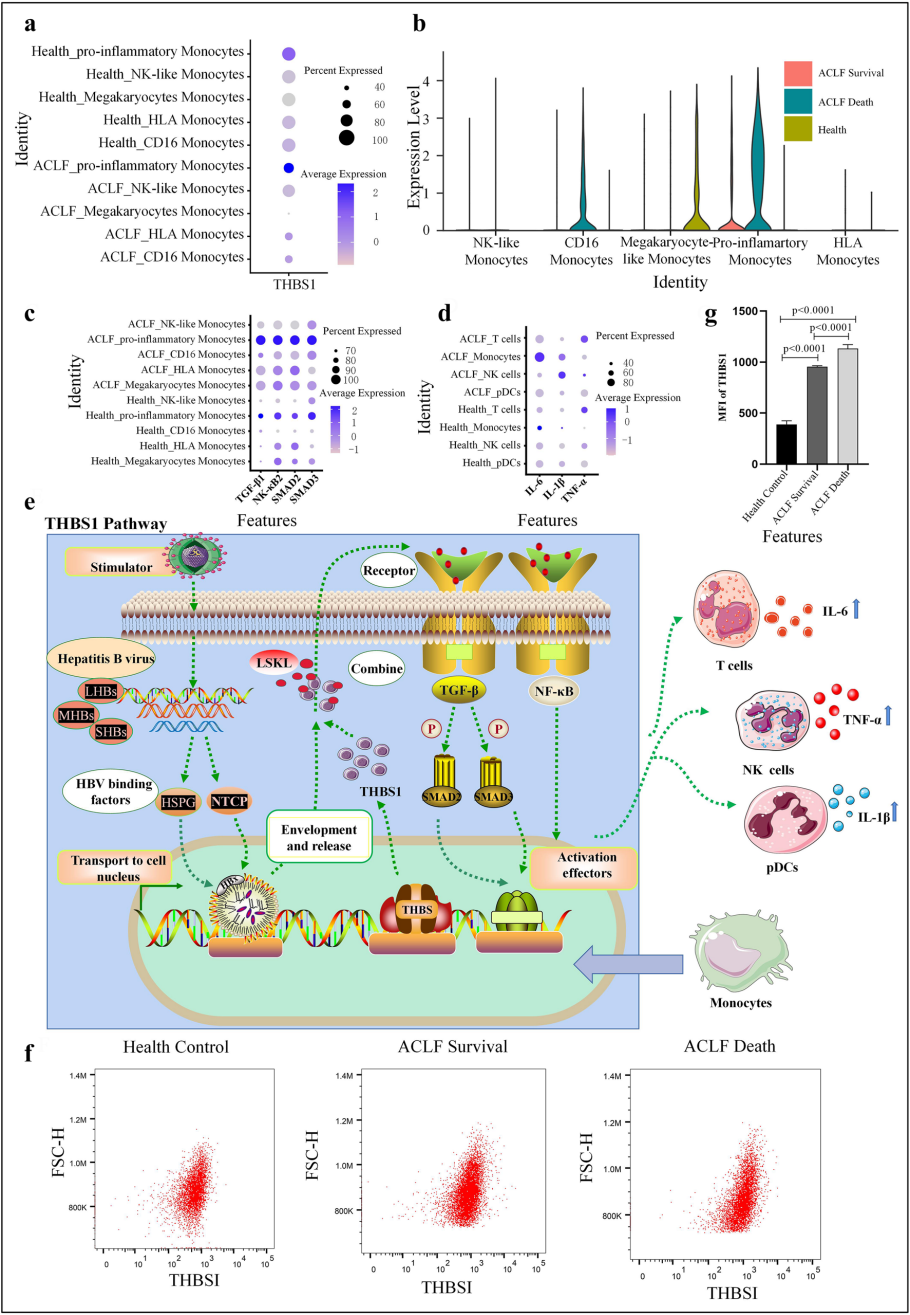
**A** Dot plot showing THBS1 expression in monocyte subpopulations in ACLF patients and healthy controls. **B** Dot plot showing THBSI expression in monocyte subpopulations in healthy controls, ACLF survival group, and ACLF death group. **C** Dot plot showing the expressions of SMAD2, SMAD3, TGF-β1, and NK-κB1 in monocyte subpopulations in ACLF patients and healthy controls. **D** Dot plot showing the expressions of IL-6, TNF-α, and IL-1β in ACLF patients and healthy controls. **E** THBS1 pathways in monocytes (the pathways and possible mechanisms by which THBS1 activates related transcription factors and produces inflammatory factors). **F** Flow cytometry shows the mean fluorescence intensity (MFI) of THBS1 in one healthy control, one ACLF survival patient, and one ACLF death patient. **G** Bars show the MFI of THBS1 in healthy controls (*n* = 3), ACLF survival group (*n* = 3), and ACLF death group (*n* = 3). *IL-6* interleukin-6, *TNF-α* tumor necrosis factor alpha, *IL-1β* Interleukin-1β

Since the pro-inflammatory monocytes showed most significant changes in gene expression in ACLF, the phenotypic changes in this subpopulation might be associated with the poor prognosis of ACLF. Accordingly, our further analysis focused on pro-inflammatory monocytes. Although clinical measures, such as creatinine (CREA) and total bilirubin (TBil), can be used to monitor disease activity and treatment response, these assessments provide little information on the immune status or underlying prognostic mechanisms of ACLF. To determine the relevant indicators of ACLF progression, we detected the expressions of thrombospondin 1 (THBS-1) in the monocytes of ACLF patients at the single-cell transcriptional level and found that the expression of THBS1 was more obvious in the pro-inflammatory monocytes of ACLF patients (Fig. 4A). Moreover, the THBS1 expression gradually increased in the order of healthy controls, ACLF survival group, to ACLF death group. Therefore, THBS1 may be a factor influencing ACLF progression and poor prognosis (Fig. 4B).

THBS1 belongs to the thrombospondin (THBS) family and has biological functions, such as promoting immune activation and enhancing inflammatory response [30]. We analyzed the expressions of potential molecules associated with the THBS1 pathway using scRNA-seq. The results showed that pro-inflammatory monocytes with high expression of THBS1 gene in ACLF also had high expressions of TGF-β, NF-κB, SMAD2 and SMAD3; in addition, the gene expressions of IL-6, IL-1β, and tumor necrosis factor-α (TNF-α) also increased (Fig. 4C and D). Previous studies have suggested that THBS1 stimulates SMAD2 and SMDA3 phosphorylation through the NF-κB signaling pathway and transforming growth factor β1 (TGF-β1) pathway and ultimately regulates the secretion of inflammatory cytokines (e.g., IL-6, IL-1β, and TNF-α) [27, 31]. Therefore, we speculate that the highly expressed THBS1 in pro-inflammatory monocytes can bind to the conserved sequence (leucine–serine–lysine–leucine [LSKL]) to activate the TGF-β and NF-κB pathways and induce the upregulation of downstream transcription factors (e.g., SMAD2 and SMAD3), which drive the secretion of IL-6, IL-1β, and TNF-α from multiple effector cells, thus leading to ACLF progression and even death (Fig. 4E).

Flow cytometry further showed that the MFI of THBS1 in monocytes was significantly higher in ACLF survival patients (962.16 ± 74.69) than in healthy controls (485.16 ± 67.32), and significantly higher in the ACLF death group (1129.18 ± 74.43) than that in the ACLF survival group (Fig. 4F and G). Therefore, high THBS1 expression may be a potential biomarker of ACLF progression and poor prognosis, and THBS1 may be used as an indicator for evaluating ACLF progression.

## Discussion

In the present study, we investigated the circulating pro-inflammatory monocytes at the single-cell level in healthy individuals and ACLF patients using scRNA-seq. Notably, we identified five monocyte subpopulations with distinct gene expression patterns in both healthy individuals and ACLF patients, providing a more precise subclassification of the existing monocyte categories. Furthermore, we found that the gene expression signatures, activation status, and upstream and downstream pathways of pro-inflammatory monocytes in ACLF patients were significantly changed compared with those in healthy controls. We also discovered that THBS1 is a specific biomarker that was preferentially enriched in the pro-inflammatory monocytes of ACLF patients.

Our knowledge of ACLF mainly derived from research on peripheral circulating immune cells. Analysis of monocytes has emphasized the importance of inflammatory response and immune mechanisms in ACLF progression [8]. A recent study showed that a strong systemic inflammatory response was a major cause of acute exacerbations in patients with ACLF, where immune cells play a key role [4]. As key immune effector cells, monocytes can sense environmental changes and present antigens to downstream cells, thus playing a central role in the initiation and resolution of inflammation. In the present study, we compared monocytes between healthy controls and ACLF patients and the five monocyte subpopulations included pro-inflammatory monocytes, CD16 monocytes, HLA monocytes, megakaryocyte-like monocytes, and NK-like monocytes according to the results of expression profiling and functional annotation of the cellular markers. Key immune factors and underlying molecular mechanisms associated with ACLF are determinants of the severity of liver injury [4]. In the present study, scRNA-seq showed that compared with the healthy control group, the ACLF group had a higher proportion of monocytes and higher levels of inflammation-related genes. During ACLF progression, monocytes trigger and drive hepatic immune system and systemic inflammatory responses by recognizing and responding to injury-associated molecules released by damaged hepatocytes. The pro-inflammatory monocytes also highly expressed *THBS1*, *PRS20*, and* IL32* genes, which are involved in viral infection, immune activation, and pro-inflammatory responses. The function of CD16 monocytes in different diseases remains controversial. Korf et al. reported that the scavenger receptors (e.g., CD163, MRC1, CD36, and Marco), growth factors (HGF), inhibitory cytokines (e.g., IL10), chemokines (e.g., CCL22), molecules involved in the phagocytosis of apoptotic cells (e.g., MERTK and TGM2) and M2-like surface phenotype (i.e., MS4A4A) were up-regulated in CD16 monocytes, did not produce reactive oxygen species (ROS) or express inflammatory chemokine receptors such as CCR2. It is indicated that CD16 monocytes had anti-inflammatory effects [8]. Kapellos et al. found that CD16 monocytes produced high levels of IL-6 when stimulated and thus were involved in the pathogenesis of inflammatory diseases [23]. In our study, the non-classical CD16 monocytes highly expressed *IFI27* and *PRS20*, which can induce inflammatory responses; meanwhile, the expression levels of IL1B, NLRP3 and CCL3 were reduced. Therefore, rather than simply promoting or inhibiting inflammation, CD16 monocytes have dual regulatory effects in apoptotic signaling pathways, inflammatory responses, and cytokine chemotaxis and migration in ACLF. Thus, different monocyte subpopulations have their unique functions in ACLF.

After the key role of pro-inflammatory monocytes in the pro-inflammatory response was confirmed, we screened potential biomarkers and identified the specific marker THBS1 in pro-inflammatory monocytes by scRNA-seq. THBS1 expression gradually increased in the order of the healthy controls, ACLF survival group, and ACLF death group, which further demonstrated the specificity of THBS1 in ACLF. Previous experiments have shown that the expression of THBS1 in the liver tissue of ACLF rats gradually increased from healthy rats to ACLF rats. THBS1 is a potential biomarker associated with ACLF-related immune imbalance. It is highly expressed in pro-inflammatory monocytes and thus may be used as a specific expression marker [4]. Studies have shown that THBS1 induces the activation and signaling of TGF-β1 and NF-κB, aggravates liver damage, promotes liver cell death, and ultimately leads to deterioration of liver function [27, 31]. In addition, monocytes can also produce a large number of inflammatory mediators through SMAD2 and SMAD3, including dendritic cell (DC)-derived TNFSF13 and IL-18 and T cell-derived IL-2 and IL-4, which can promote the survival, proliferation, and differentiation of B cells [30]. THBS1 can induce the up-regulation of IL-1β, IL-6, and TNF-α in immune cells, among which IL-6 and TNF-α play an important role in the immune response because they not only can transmit signals but also regulate cell differentiation and apoptosis by activating inflammatory cells. In addition, IL-6 and TNF-α can mediate the activation of T cells and B cells to produce granulocyte–macrophage colony-stimulating factor (GM-CSF), which promotes the proliferation and activation of monocytes to further induce the high expression of IL-6, which can accelerate the pro-inflammatory response after bacterial infection, resulting in an cytokine storm. Therefore, a better understanding of the mechanism of action of THBS1 and its exact function in regulating the inflammatory cytokine profile are critical for the development of novel, more effective therapeutic approaches for ACLF.

In conclusion, based on the unbiased scRNA-seq method, we constructed the circulating monocytes maps and supplemented the immune map of ACLF. By subdividing the monocyte subpopulations, we revealed that pro-inflammatory monocytes were closely related to ACLF progression, and therapies targeting pro-inflammatory monocytes may be a promising approach to blocking ACLF progression. We also discovered that THBS1 is a specific biomarker that was preferentially enriched in the pro-inflammatory monocytes of ACLF patients; thus, high THBS1 expression may be suggestive of ACLF progression and poor outcomes. However, the present study was limited by its small sample size, and future longitudinal studies with larger sample cohorts will be more helpful in investigating the survival and prognosis of ACLF patients. The novel biomarkers discovered in this study may advance our understandings of the mechanisms of monocytes and add potential clinical value for the targeted therapy of ACLF.

## Supplementary Information

Below is the link to the electronic supplementary material.

Supplementary file1 (XLSX 12 KB)

## Data Availability

The scRNA-seq data reported in this paper have been stored in the Genome Sequence Archive (GSA) in National Genomics Data Center (Accession No. HRA002467). All research data are included in this article.
